# Complications of Mesenteric Vein Thrombosis: Heterozygous Factor V Leiden Mutation Leads to Pulmonary Embolism in a Patient With Post–Bowel Resection Surgery

**DOI:** 10.1002/ccr3.70092

**Published:** 2025-01-06

**Authors:** Hafiz Muhammad Hamza, Muhammad Muiz Malik, Ayaz Ahmed Awan, Muhammad Daoud Tariq, Mohamed Daoud, Aman Goyal

**Affiliations:** ^1^ Department of Internal Medicine Foundation University Medical College Islamabad Pakistan; ^2^ Department of Internal Medicine Bogomolets National Medical University Kyiv Ukraine; ^3^ Department of Internal Medicine Seth GS Medical College and KEM Hospital Mumbai India

**Keywords:** bowel resection, factor V, Leiden mutation, mesenteric vein, pulmonary embolism

## Abstract

It is critical to recognize pulmonary embolism as soon as possible in patients who have gastrointestinal problems pre‐ and post‐surgery. Even in the absence of conventional risk factors, the Factor V Leiden mutation emphasizes the importance of a thorough thrombophilia assessment. To effectively manage and prevent thrombotic episodes, prompt anticoagulant medication and genetic screening for family members are essential.

## 1 Introduction

Venous thromboembosis (VTE), that constitutes deep vein thrombosis (DVT) and pulmonary embolism (PE), is a serious public health dilemma because of its high rate of morbidity and mortality that requires hospitalization [1]. The main causes of VTE are pregnancy, estrogen medication, trauma or fracture, immobilization, surgery, and aggressive cancer. There are also genetic risk factors, the most common of which is the Leiden mutation in factor V, which constitutes approximately 2%–4% of the global population, with an annual incidence of 0.1% and in 20% of VTE cases [2]. Because activated protein C (APC) cleaves factor Va and factor VIIIa, deactivated factor V is less functional as a cofactor, and these two modifications in the coagulation mechanism increase the risk of thrombosis overall. Factor V Leiden mutation is a point variant that abolishes an explicative cleavage location in factor V and factor Va, making operative factor V impervious to dysfunction by APC [3]. Due to its inability to cleave at Arg 506, inactivated factor V cannot serve as a cofactor in this matter of mortification of factors Va and VIIIa. Consequently, the anticoagulant effect of factor V is diminished, increasing the risk of venous thrombosis [3]. Both the frequency of mesenteric vein thrombosis and the number of cases that are unintentionally detected have increased as a result of advancements in diagnostic methods. Between 1970 and 1982, the estimated incidence was 2 per 100,000, and between 2000 and 2006, it was 2.7 per 100,000. 1/1000 ER admissions and 6%–9% of all acute mesenteric ischemia cases are caused by mesenteric vein thrombosis. The mean age of patients is between 45 and 60 years and at presentation, there is a little male to female predominance [4].

Given that factor V is impervious to being inactivated by functional protein C, the mutation in factor V impairs factor V's ability and its function as an anticoagulant. As a result, factors Va and VIIIa degrade less, which increases the risk of thrombotic events for the person. The significance of focused therapies in at‐risk groups is highlighted by the comprehension of the molecular process behind the leiden mutation in factor V mutation and its impact on venous thromboembolism.

## 2 Case Presentation

### 2.1 Case History and Examination

An Asian male, 60 years old from Rawalpindi, was admitted to the critical care unit for acute breathlessness at recess accompanied with palpitations and presented with a history of chronic epigastric pain and constipation persisting for several months. Four months prior to presentation, he underwent bowel resection surgery due to complications associated with his gastrointestinal symptoms. Postoperatively, he required a colostomy bag for bowel elimination. Despite the surgical intervention, he reported ongoing epigastric discomfort and constipation. Additionally, he described new symptoms of shortness of breath and tachycardia. Moreover, there was no parental history of thromboembolic malady. He had smoked tobacco for 20 pack years in the past. Even if he stopped smoking 2 years ago, his past medical history can still have an impact on his general health and recuperation after surgery. Upon examination, he appeared anxious and in distress. Vital examination disclosed a heart rate of 107 beats per minute and a respiratory rate of 24 breaths per minute, indicative of tachycardia and tachypnea, respectively. His blood pressure was within normal limits. Abdominal examination revealed a well‐healed surgical incision and the presence of a colostomy bag without signs of peritonitis or bowel obstruction. He was of standard weight and height with a body mass index of 23.3 kg/m^2^ and an O_2_ saturation of 89% on room atmospheric pressure. The laboratory investigations of patient along with their reference ranges are given in Table 1. The pleuropulmonary analysis was normal, and there were no indications of heart dysfunction on the cardiovascular or left side. The accompanying physical examination was non‐specific. All the causes of acute dyspnea, such as asthma exacerbation, pneumothorax, cardiac tamponade, and acute lung disease, were included in the differential diagnosis.

**TABLE 1:** Laboratory investigations.

Test	Result	Reference range
Lupus anticoagulant	< 1.0	< 1.2 (ratio)
Factor v Leiden mutation	Detected (heterozygous)	
Anti–cardiolipin antibody (IgG)	1.8	< 10 GPL‐U/mL
Anti–cardiolipin antibody (IgM)	1.6	< 7 MPL‐U/mL
Homocysteine	10.6	< 16.2 umol/L
D‐dimer	4927	< 230 ng/mL
Uric acid	7.3	3.4–7.2 mg/dL
Blood urea	29	10–50 mg/dL
Serum blood urea nitrogen	14.4	6–23 mg/dL
Serum creatinine	0.73	0.8–1.3 mg/dL
eGFR	116	> 60 mL/min/1.73 m
Total bilirubin	0.3	0.1–1.1 mg/dL
ALT	31	5–55 U/L
AST	28	9–40 U/L
ALP	100	30–115 U/L
Gamma GT	61	< 55 U/L
Overall protein	9.2	6–8 g/dL
Globulin antibody	4.2	2.5–3.5 g/dL

As a consequence of the severity of the pulmonary embolism, the patient underwent an etiological workup throughout his hospital stay, which included an immunological workup and tumor markers. At the lower limb venous Doppler ultrasonography level, there was no evidence of DVT bilaterally. On performing arterial Doppler, there was mild age‐related atherosclerotic intimal calcifications in the common femoral, superficial femoral, and origin of the profunda femoris arteries bilaterally which was suggesting mild peripheral vascular arterial disease. However, there was no significant luminal narrowing. Additionally, a thrombophilia evaluation that objectified a heterozygous mutation of factor V of Leiden had helped him.

### 2.2 Treatment, Outcome, and Follow‐Up

**FIGURE 1:**
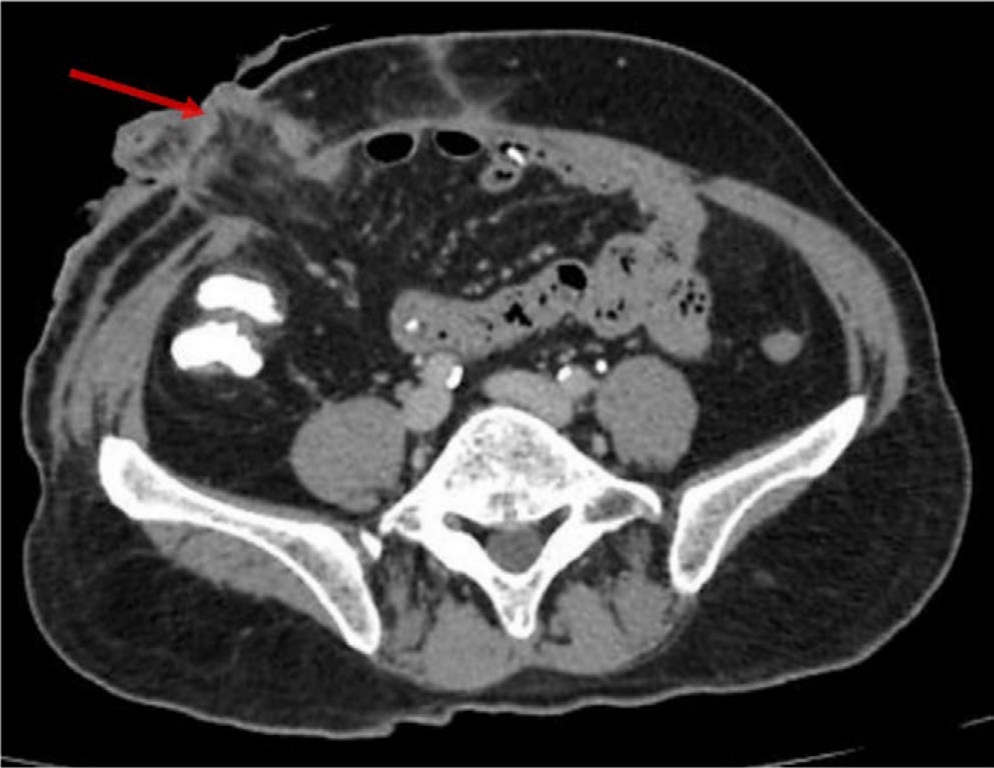
Visualization of ileostomy in CTPA imaging.

**FIGURE 2:**
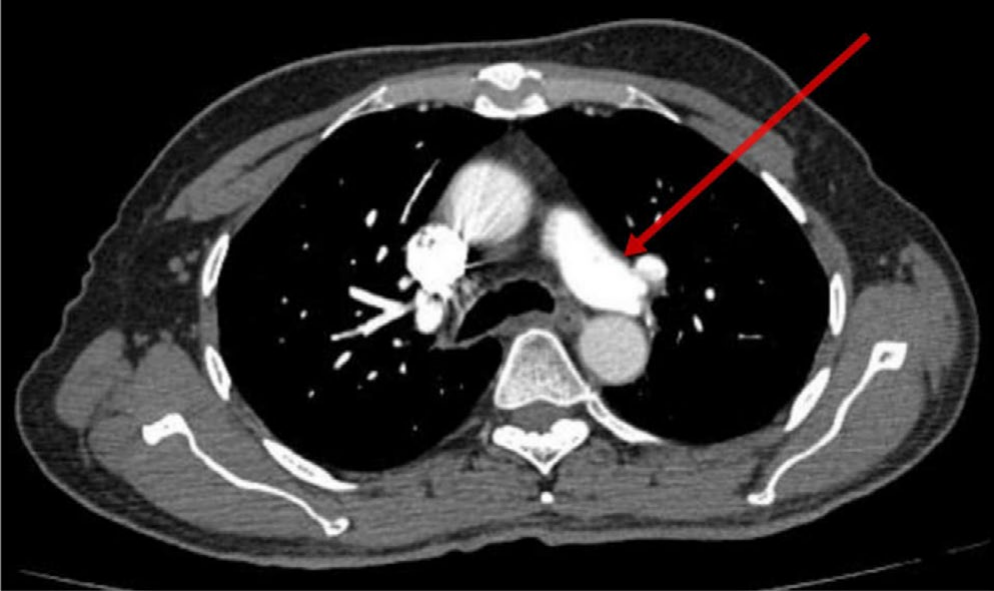
Visualization of left pulmonary arteries in CTPA imaging.

**FIGURE 3:**
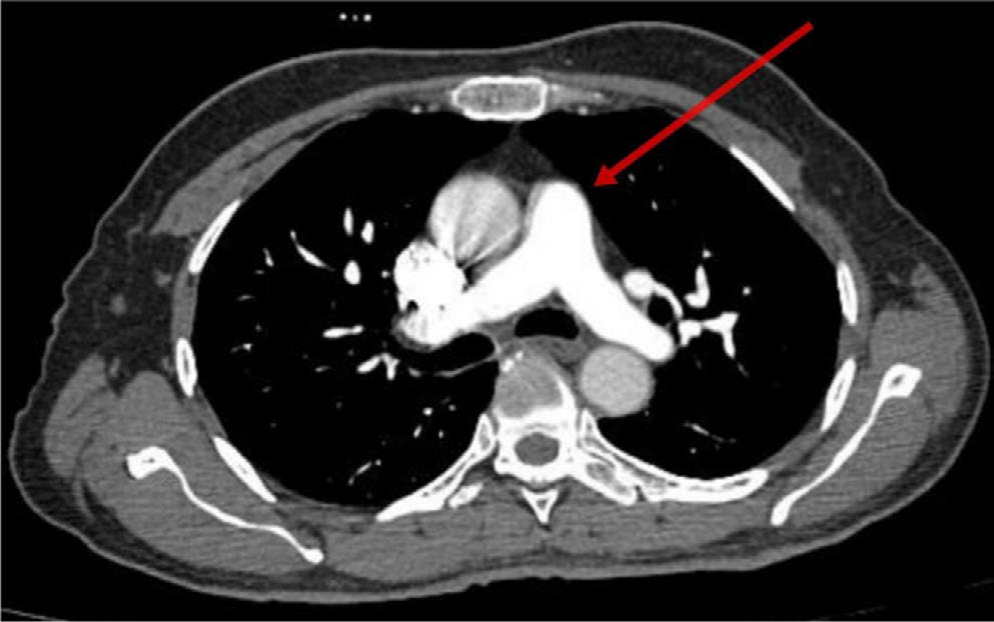
Visualization of the main pulmonary trunk in CTPA imaging.

**FIGURE 4:**
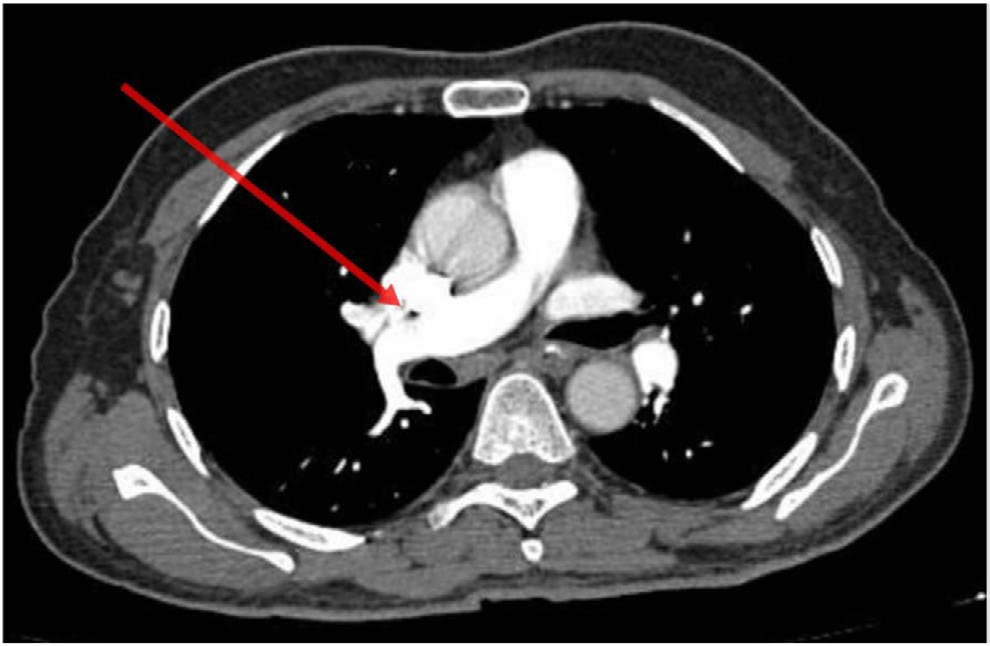
Visualization of the right pulmonary artery in CTPA imaging.

After being admitted to the cardiology critical care unit, the patient underwent conditioning and scoping, and a pulmonary embolism was diagnosed on the basis of computed tomography pulmonary angiography (CTPA). There was bilateral thickening noted along with chronic fibronodular changes bilaterally in upper lobes. Few tiny calcific foci were noted in bilateral lungs. Multiple small air containing cyst were noted in bilateral lungs. Few small filling defects were noted in segmental and subsegmental branches of basal segments of bilateral lower lobes. He was managed initially with rivaroxaban at a dose of 15 mg two times a day for 21 days then continued 20 mg one time a day. It should be mentioned that the patient stayed stable during his therapy, and on the sixth day of his hospital stay, his dyspnea went away. Thereafter he was discharged and advised to continue treatment. He was directed to the genetic medicine department for potential investigation and a screening for the Leiden mutation in factor V in first degree relatives within the family. Figure 1 depicts visualization of ileostomy because the patient still does not have colostomy reversal in CTPA imaging. Figure 2 depicts improved filling defects in segmental branches while Figures 3 and 4 depict the pulmonary trunk, right and left pulmonary arteries are normal in caliber. These images show the course of treatment and its effect during the patient's management. Each figure illustrates key stages of intervention and response to therapy, providing visual insights into the management of the case.

## 3 Discussion

One of the procoagulant factors in the clotting cascade is factor V. Functional factor V (FVa) and activated factor X (FXa) convert prothrombin to thrombin, the active form, which finally causes a clot to form. The natural anticoagulant activated protein C then breaks down FVa by cleaving it at arginine R‐506, R306, and R679 in the heavy chain [5]. A mutant gene might be inherited homozygous or heterozygous. A higher risk of developing venous thromboembolism exists in both forms [6]. Mesenteric venous thrombosis (MVT) almost invariably affects the blood arteries that drain the ascending and transverse colons rather than only the descending and sigmoid colons, is the rare cause of it (< 5%) [7]. Colon ischemia is produced by three main mechanisms: MVT, thrombotic arterial occlusion from embolic events or ruptured atherosclerotic plaque, and hypoperfusion damage, which usually affects individuals with underlying vascular disease or risk factors [8]. Compared to our patient, the mean age of the Factor V Leiden homozygous present with thrombosis is 31, which is much younger [9]. A study conducted in which congenital thrombophilia, which includes factor V mutation, antithrombin III (ATIII) derangement, protein C derangement (PCD), and protein S deficiency (PSD), was identified in 7.1% of PE patients. Remarkably, the most widespread subtype of hereditary thrombophilia is PSD (3.0%), which is trailed by PCD (2.8%) and ATIII deficiency (1.1%) [10]. The diagnosis of a factor V Leiden heterozygous mutation was made in just 1 patient (0.2%). The current guidelines advise anticoagulant treatment whether or not congenital thrombophilia is present, even though it does not manifest to raise the risk of relapse [10]. The mainstay of treatment for venous thromboembolism is anticoagulation, which is advised for at least 3 months in the majority of patients with pulmonary embolism and/or deep vein thrombosis [11]. Anticoagulant therapy aims to minimize the risk of long‐term sequelae, including chronic thromboembolic pulmonary hypertension, exertional dyspnea, and post–thrombotic syndrome, as well as to prevent recurrence and avoid fatal PE [11].

## 4 Conclusion

This case highlights the complexity of diagnosing pulmonary embolism in patients with underlying gastrointestinal issues and postoperative complications. Despite the absence of typical risk factors, like family history of thromboembolic disease or significant DVT, the presence of the Leiden mutation in factor V underscores the importance of thorough thrombophilia assessments. Prompt diagnosis and initiation of appropriate anticoagulant therapy, such as rivaroxaban, proved effective in managing the patient's symptoms and stabilizing his condition. Additionally, the necessity of genetic exploration and family screening for factor V Leiden mutation in first‐degree relatives emphasizes the significance of identifying hereditary predispositions to thrombotic events for preventive management.

## Author Contributions

**Hafiz Muhammad Hamza:** conceptualization, data curation, methodology, writing – original draft. **Muhammad Muiz Malik:** data curation, resources, writing – original draft. **Ayaz Ahmed Awan:** data curation, resources, writing – original draft. **Muhammad Daoud Tariq:** conceptualization, data curation, project administration, writing – original draft. **Mohamed Daoud:** project administration, writing – review and editing. **Aman Goyal:** conceptualization, project administration, writing – review and editing.

## Consent

Written informed consent was obtained from the patient to publish this report in accordance with the journal's patient consent policy.

## Conflicts of Interest

The authors declare no conflicts of interest.

## Data Availability

Data pertaining to this case can be obtained by contacting the corresponding author.
